# Epicardial Fibrosis Explains Increased Endo–Epicardial Dissociation and Epicardial Breakthroughs in Human Atrial Fibrillation

**DOI:** 10.3389/fphys.2020.00068

**Published:** 2020-02-21

**Authors:** Ali Gharaviri, Elham Bidar, Mark Potse, Stef Zeemering, Sander Verheule, Simone Pezzuto, Rolf Krause, Jos G. Maessen, Angelo Auricchio, Ulrich Schotten

**Affiliations:** ^1^Department of Physiology, Maastricht University, Maastricht, Netherlands; ^2^Center for Computational Medicine in Cardiology, Institute of Computational Science, Università della Svizzera Italiana, Lugano, Switzerland; ^3^Maastricht University Medical Centre, Maastricht, Netherlands; ^4^Inria Bordeaux – Sud-Ouest Research Centre, Talence, France; ^5^IMB, UMR 5251, Université de Bordeaux, Talence, France; ^6^IHU Liryc, Electrophysiology and Heart Modeling Institute, Foundation Bordeaux Université, Bordeaux, France; ^7^Fondazione Cardiocentro Ticino, Lugano, Switzerland

**Keywords:** atrial fibrillation, computer models, fibrosis, transmural conduction, EED, breakthrough waves

## Abstract

**Background:**

Atrial fibrillation (AF) is accompanied by progressive epicardial fibrosis, dissociation of electrical activity between the epicardial layer and the endocardial bundle network, and transmural conduction (breakthroughs). However, causal relationships between these phenomena have not been demonstrated yet. Our goal was to test the hypothesis that epicardial fibrosis suffices to increase endo–epicardial dissociation (EED) and breakthroughs (BT) during AF.

**Methods:**

We simulated the effect of fibrosis in the epicardial layer on EED and BT in a detailed, high-resolution, three-dimensional model of the human atria with realistic electrophysiology. The model results were compared with simultaneous endo–epicardial mapping in human atria. The model geometry, specifically built for this study, was based on MR images and histo-anatomical studies. Clinical data were obtained in four patients with longstanding persistent AF (persAF) and three patients without a history of AF.

**Results:**

The AF cycle length (AFCL), conduction velocity (CV), and EED were comparable in the mapping studies and the simulations. EED increased from 24.1 ± 3.4 to 56.58 ± 6.2% (*p* < 0.05), and number of BTs per cycle from 0.89 ± 0.55 to 6.74 ± 2.11% (*p* < 0.05), in different degrees of fibrosis in the epicardial layer. In both mapping data and simulations, EED correlated with prevalence of BTs. Fibrosis also increased the number of fibrillation waves per cycle in the model.

**Conclusion:**

A realistic 3D computer model of AF in which epicardial fibrosis was increased, in the absence of other pathological changes, showed increases in EED and epicardial BT comparable to those in longstanding persAF. Thus, epicardial fibrosis can explain both phenomena.

## Introduction

The progression of atrial fibrillation (AF) is mediated by ion-channel remodeling and structural alterations including fibrosis (Schotten et al., 2011). Both processes increase the likelihood and complexity of fibrillation. Recent studies have also shown an increased dissociation of activation between the epicardial layer and the endocardial bundle network, and an elevated incidence of epicardial breakthroughs (BTs) in later stages of AF (Allessie et al., 2010; Eckstein et al., 2011; Verheule et al., 2013; de Groot et al., 2016).

Theoretically, this endo–epicardial dissociation (EED) and high incidence of BTs could be explained by loss of electrical coupling between the epicardial layer of the atrial wall and the endocardial bundle network (Gharaviri et al., 2017). However, Verheule et al. (2013, 2014) demonstrated that the endomysial fibrosis that accompanies the transition from persistent to permanent AF in goats occurs almost exclusively within the epicardial layer, particularly in the outer 1 mm of the atrial wall, while endocardial bundles remain unaffected. It is not obvious and experimentally difficult to test whether this preferentially epicardial distribution of fibrosis suffices to explain the observed increase in EED and BTs or whether other mechanisms underlie these phenomena. Therefore, computer modeling can be useful to investigate this question.

To test the hypothesis that epicardial fibrosis causes EED and BTs, we simulated AF in a highly detailed computer model capable of three-dimensional conduction in the atrial wall. This model included an endocardial bundle network, an epicardial layer with varying wall thickness, and realistic layered fiber orientations. We investigated the effect of epicardial fibrosis, fibrosis within the outer muscular layer of epicardial tissue, on EED and BTs, and qualitatively compared its effects with the results of simultaneous direct contact high-resolution endo–epicardial mapping in human right atria (RA).

## Materials and Methods

### Computational Model of AF

**FIGURE 1:**
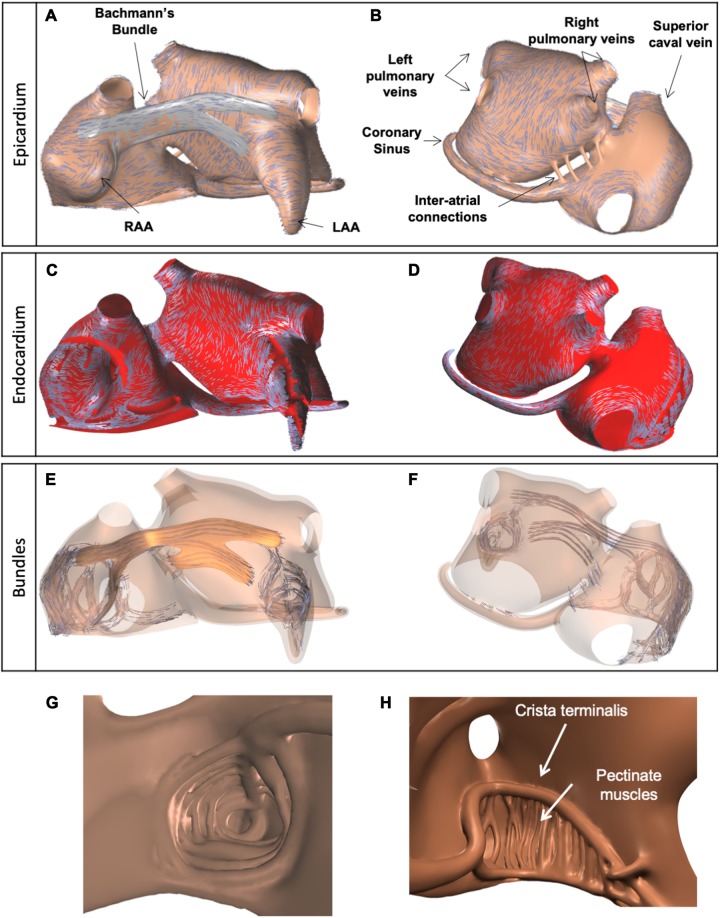
Visualization of anatomical model of the atria, used for simulations, and three layers of fiber orientations. **(A)** Anterior view of the model and epicardial layer of fiber orientations, indicated by blue lines. **(B)** Posterior view of the model, with epicardial layer of fiber orientations. **(C,D)** Endocardial layer of fiber orientations. **(E,F)** Fiber orientations in endocardial and epicardial bundles, including BB (orange). **(G)** Trabecular network of the LAA. **(H)** Trabecular network of the RA, with 20 pectinate muscles (interior wide-angle view).

Magnetic resonance imaging (MRI) data of a subject with a normal atrial anatomy were used to reconstruct global atrial shape. The endocardium was traced manually and extended to form a “myocardial envelope,” a closed surface inside which atrial myocardium could be present. During the final mesh construction the actual myocardium was defined algorithmically by filling this surface up to 1 mm from the endocardium for the right atrium and up to 2 mm for the left, based on the results of Wang et al. (1995) and Ho et al. (1999). To achieve these thicknesses (in a hexahedral mesh with 0.2 mm edge lengths) we used a distance transform with chamfer distances of 1, $\sqrt{2}$, and $\sqrt{3}$, to define the distance from the cavity and retained the elements with less than the target distance. Based on anatomical studies (Ho et al., 2002; Ho and Sanchez-Quintana, 2009), bundle structures including 20 pectinate muscles (PM), Bachmann’s Bundle (BB), interatrial bundles, and the crista terminalis (CT) were added manually using Blender (The Blender Foundation, Amsterdam, Netherlands) as a 3D editing tool (Potse et al., 2016). Endocardial bundles were created by drawing flexible tubes with variable cross sections just below the endocardium. Part of the endocardial bundles were in contact with the epicardial layers, others were freely running through the cavity of the atria. BB was defined by creating a closed surface just outside the myocardium of the two atria. The model contained left and right atrial appendages (LAA and RAA), an LAA trabecular network, and the coronary sinus (CS) musculature (Figure 1). The BB connected regions between the superior caval vein and RAA with the superior wall of the left atrium (LA) (Figure 1A). A few narrow fiber tracts connected the CS to the posterior wall of the LA, following the anatomical study by Chauvin et al. (2000).

One to three layers of fiber orientation were embedded in the model based on the work of Ho et al. (2002) and Ho and Sanchez-Quintana (2009) (Figure 1). The fiber orientations were defined by drawing several sets of splines on top of the atrial walls and BB. For the left atrial wall, two sets of splines were used to assign different fiber orientations to the endocardial and epicardial halves of the wall, in order to represent the septopulmonary and septoatrial bundles (Ho et al., 2002). Another set of splines was used to define a circumferential fiber orientation in the pulmonary and caval vein ostia. A final set of splines was used for BB.

From the manually created objects we built a computational mesh consisting of hexahedral elements with 200 μm sides. This was done using dedicated software. Each model element had its own tissue type and fiber orientation. Elements were created within the myocardial envelope (up to the defined thicknesses of 1 mm in the RA and 2 mm in the LA) and bundles. Elements within bundles had type “wall” and a fiber orientation aligning with the bundle axis. Elements within BB had type “BB” and a fiber orientation aligning with the nearest spline. Elements within the left atrial wall had type “wall” and a fiber orientation aligning with the nearest spline of the appropriate layer. Elements in the right atrial wall also had type “wall” and obtained an orientation perpendicular to the nearest endocardial bundle and parallel to the epicardial surface. A small number of right-atrial elements located more than 1 cm away from any bundle (mostly in the intercaval area) had no fiber orientation and were assigned type “iso” for isotropic conductivity. The algorithmically determined three layers of fiber orientations are illustrated in Figure 1. Comparison with results of a recently published submillimeter-resolution diffusion tensor MRI study (Pashakhanloo et al., 2016) showed good agreement of the prevailing local fiber orientations in our model with human atria.

The material properties for the atria were set to produce an approximately normal P wave in case of sinus rhythm (SR). The effective monodomain conductivities along and across the fiber are given in Table 1. The tissue surface to volume ratio was 800 cm^–1^ throughout the atrial myocardium.

**TABLE 1:** Tissue conductivity parameters (σ) used in the simulations.

Material	σ_*iL*_	σ_*iT*_	σ_*iC*_	σ_*eL*_	σ_*eT*_	σ_*eC*_	*G*_*mL*_	*G*_*mT*_	*G*_*mC*_
Wall	3.0	0.3	0.3	3.0	1.2	1.2	1.5	0.24	0.24
Iso	1.5	1.5	1.5	1.5	1.5	1.5	0.75	0.75	0.75
BB	9.0	0.3	0.3	9.0	1.2	1.2	4.5	0.24	0.24
Fibrotic	3.0	0.0	0.0	3.0	1.2	1.2	1.5	0	0

*The units are mS/cm. The subscript “i” stands for intracellular, “e” for extracellular, “L” for longitudinal, “T” for transverse (within a tissue sheet), and “C” for across-sheet.*

The model geometry consisted of approximately 5 million nodes. The simulations were performed with the propag-5 software (Potse et al., 2006; Krause et al., 2012) and run on a Cray XC50 supercomputer.

Electrical activity was simulated with a monodomain reaction–diffusion equation:

(1) $$\left\{ \begin{array}{c} C_{\text{m}}\,\partial_{t}V_{\text{m}}\,\left(x,t\right)=\beta^{-1}\,\nabla \cdot \left[G_{\text{m}}\,\left(x\right)\,\nabla V_{\text{m}}\,\left(x,t\right)\right]-I_{\text{ion}}\,\left(V_{\text{m}},y\right)\\ \partial_{t}\text{y}\,\left(x,t\right)=\text{F}\,\left(V_{\text{m}}\,\left(x,t\right),\text{y}\,\left(x,t\right)\right) \end{array}\right.$$

where *C*_*m*_ is the membrane capacitance (set to 1 μF/cm^2^), *V*_*m*_ the transmembrane potential, *G*_m_ = *G*_i_(*G*_i_ + *G*_e_)^−1^*G*_e_ the monodomain conductivity tensor field, *y* the state vector of the ionic model, *I*_*ion*_ a function describing the total transmembrane ionic current density, and *F* a function describing the temporal evolution of the ionic model.

Membrane dynamics (*I*_*ion*_ and *F*) for each model node were described by the Courtemanche–Ramirez–Nattel model (Courtemanche et al., 1998) with minor numerical adaptations for stability (Potse, 2019). Simulations were performed with a second-order accurate finite-difference method (Potse et al., 2006) on a hexahedral mesh at 0.2 mm resolution. Differential equations for potentials and ion concentrations were integrated with the forward Euler method and gating variables with the Rush–Larsen method (Rush and Larsen, 1978) using a time step of 0.01 ms. Boundary conditions were **n**⋅(*G*_m_∇⁡*V*_m_) = 0 on the boundary of the myocardium where the normal vector *n* is determined from the fiber angles. The implementation of the boundary conditions is implicit in the formulation of Saleheen and Ng (1997). To incorporate changes in ionic currents as observed in AF, conductances for the transient outward current (*I*_to_), calcium current (*I*_Ca,L_), and inward rectifier potassium current (*I*_K__1_) were set at 40, 35, and 200% of their normal values, respectively (Gharaviri et al., 2017).

### Fibrosis

Fibrotic tissue was modeled by assigning the special type “fibrotic” to a subset of voxels in the computational model. The “fibrotic” voxels were conductive with zero conductivity across the fiber orientation, and normal conductivity along the fibers. This method was chosen to represent the primarily lateral uncoupling found by Spach and Dolber (1986) in fibrotic atrial tissue.

**FIGURE 2:**
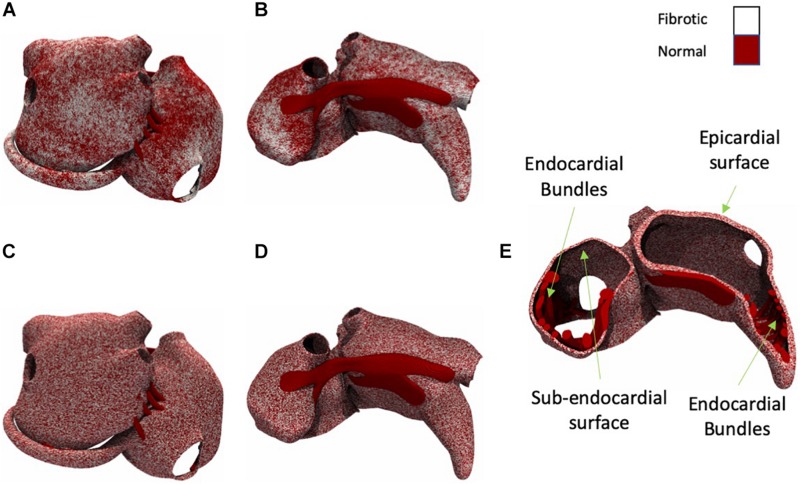
Posterior and anterior view of the atria with patchy and uniform fibrosis (white shows fibrotic tissue and dark red shows normal tissue). **(A)** Moderate fibrotic model with patchy fibrosis pattern (posterior view). **(B)** Moderate fibrotic model with patchy fibrosis pattern (anterior view). **(C)** Moderate fibrotic model with uniform fibrosis pattern (posterior view). **(D)** Moderate fibrotic model with uniform fibrosis pattern (anterior view). **(E)** A cross-section view of the atria with uniform fibrosis.

We have developed algorithms that produce fibrosis patterns similar to those obtained from LGE-MRI (McDowell et al., 2012; Akoum et al., 2013) but in a higher resolution (Figures 2A,B). Since the spatial distribution of fibrosis affects propagation (Verheule et al., 2004), we have compared patchy (Figures 2A,B) and uniform patterns (Figures 2C,D). We quantified their effects on the AF conduction pattern complexity, EED, and BT prevalence. The algorithm to simulate fibrosis proceeded as follows. For patchy fibrosis patterns, we generated a spatially correlated, anatomy-tailored random field as described in detail in the Supplementary Material and the previous studies (Pezzuto et al., 2018, 2019). The random field was the superposition of two random fields with correlation lengths of 4 mm and 2 cm. Secondly, for each of the model’s 5 million elements, the random field (scaled between zero and one) defined the probability for the element to be fibrotic. The threshold on the random field for defining fibrotic tissue was set such that the total fibrotic volume was equal to a given fraction of the total atrial tissue. The result of the procedure was a heterogeneous (patchy) distribution of the fibrosis (for details see the Supplementary Material). For uniform fibrosis, the procedure was the same, but the probability was set uniform across the tissue. Fibrosis distributions generated using this method span from the epicardial layer to subendocardial layer but not to the endocardial muscle bundles (Figure 2E).

Both uniform and patchy fibrosis simulations were performed with control, slight, moderate, and severe fibrotic degrees, in which 0, 50, 70, and 80% of segments were fibrotic.

### AF Initiation

**FIGURE 3:**
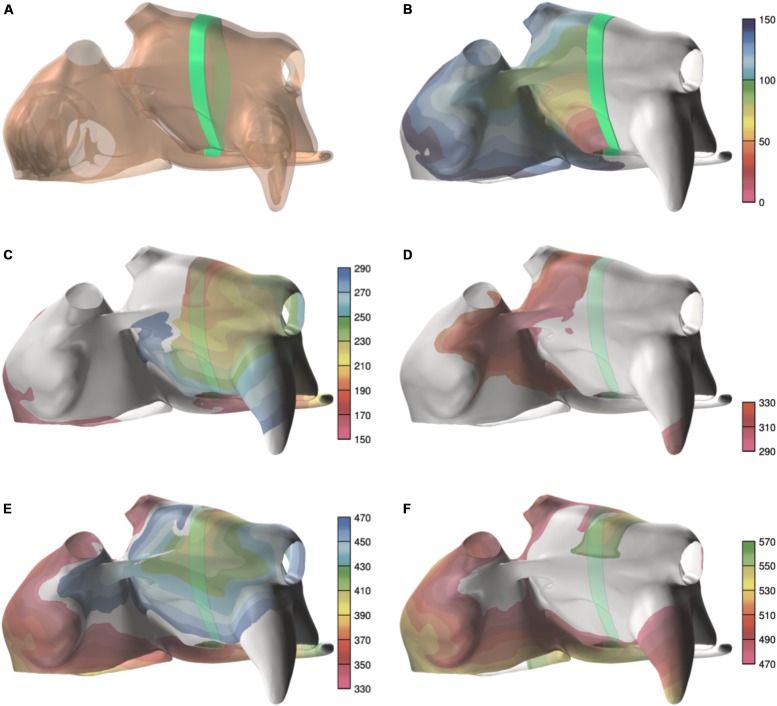
Example of a spiral wave initiation. **(A)** Temporary line of block (green line). **(B)** Activation during the first 150 ms, after pacing near the mitral ring, with the block line in place. **(C)** The next 140 ms, after the block line was removed. Activation first crosses the former block line in the area between the superior pulmonary veins. **(D–F)** The next two cycles of the reentry. The colorbar indicates the activation time, and the scales are in milliseconds.

To initiate AF, a single pacing site in combination with an associated temporary block line was used (Figures 3A–F). Temporary block was implemented by setting **F**(*V*_m_,**y**) = 0 in Eq. 1 for a given region. To exclude bias resulting from preferential conduction patterns resulting from a certain pacing site, 10 episodes of AF were initiated by pacing at 10 different locations for each group.

### Analysis

#### Detection of Waves in Simulations

A wave was defined as a contiguous volume in which all nodes have transmembrane voltages above a threshold of −60 mV. Waves size is quantified as the number of nodes in this contiguous volume. The number of waves and Wave size were calculated at each millisecond of simulated time.

#### Detection of Breakthroughs in the Simulations

A BT was defined as a wave that appears in the epicardial layer and cannot be related to the propagation of other waves in this layer. To detect BTs, wave sizes at each time step were calculated. If a wave smaller than a threshold of nine nodes appeared within one layer and this wave had an overlap with a wave in the opposing layer, it was considered as a BT candidate. Each BT candidate was monitored and if its size increased by at least a factor 2 within 10 ms it was confirmed as a BT.

#### Simultaneous Endo–Epicardial Mapping in the Patients

Seven patients, four with longstanding persistent AF (persAF) and three without a history of AF (SR group), were included. All patients underwent on-pump cardiac surgery through median sternotomy at Maastricht University Hospital, Maastricht, Netherlands. In patients without AF, the arrhythmia was acutely induced by rapid pacing. The local ethical committee approved this study and written informed consent was obtained from each patient.

**FIGURE 4:**
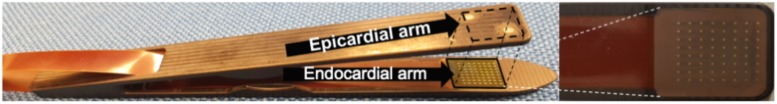
The endo–epicardial electrode contains two identical plaques of 8 × 8 unipolar electrodes.

Simultaneous endo–epicardial mapping of the RA was performed as described previously (Eckstein et al., 2011; Verheule et al., 2014). In short, we developed a tongs-shaped mapping device (Figure 4) with both arms ending in a square plaque containing 64 unipolar electrodes (8 × 8; inter-electrode distance 1.5 mm), directly facing each other when approximated. During surgery, before cannulation of the RA, the endocardial arm of the tongs electrode was introduced into a purse-string suture. All recordings were performed in the trabeculated part of the RA. In patients in SR, AF was induced through burst pacing by epicardial pacing wires. When AF was achieved and maintained for 30 s, the arms of the electrode were approximated to both sides of the atrial wall and a recording was started. By design, the electrodes were parallel at 3 mm distance. However, the surgeon could adjust the distance depending on the quality and amplitude of the electrogram signals. Unipolar electrograms were recorded at a sampling rate of 1 kHz using a band-pass filter of 0.5–500 Hz.

#### Local Activation Detection in Patient Recordings and Simulations

To detect local activation time we used automated algorithms described previously (Zeemering et al., 2012). Local deflections were detected after filtering out baseline drift and cancelation of ventricular far fields. Negative deflections in the electrograms were detected using a template matching procedure. Deflections representing local activations were identified by optimizing the match of the obtained intervals to the AF cycle length (AFCL) probability density function using a probabilistic algorithm (Zeemering et al., 2012).

In simulations, activation times were defined as the instant of steepest upstroke in simulated action potentials (APs) for each model node. From these activation times, we calculated the AFCL and the local conduction velocity (CV). Fibrillation waves were defined as groups of local activations surrounded by lines of block (Zeemering et al., 2012). The number of fibrillation waves and the number of BTs were calculated separately for the endocardial and epicardial layers.

#### Calculation of EED of Electrical Activity

In human recordings, the epicardial and endocardial electrodes were directly facing each other, allowing us to quantify activation time differences for each opposing electrode pair.

To calculate EED in simulations, model nodes were categorized as epicardial, endocardial, or endocardial bundle. Each endocardial bundle node was paired with the nearest epicardial node.

In both clinical and *in silico* recordings, activation time differences across the wall were used to assess the degree of EED. The smallest activation time differences between each electrode and either the directly opposing electrode or one of the eight electrodes surrounding the opposing electrode were plotted in histograms of endo–epicardial activation time differences. The degree of EED was determined from these histograms as previously described (Eckstein et al., 2011). Briefly, two populations of activation time differences representing dissociated and non-dissociated activity were identified by fitting a two-component Gaussian function to the histogram (Eckstein et al., 2011).

### Statistics

All human and simulation data are expressed as means with standard errors (±SE). For human data, we used a mixed model analysis to test for differences between wave characteristics in the endo- and epicardial planes in AF and SR groups.

For simulation data, statistical tests were performed to compare four groups of simulations (control, slight, moderate, and severe fibrotic). The average number of waves, BTs, and EED percentage during the whole simulation period were calculated for each individual simulation. The results of the four groups were compared using one-way ANOVA with a Bonferroni correction.

All statistical analyses were performed using SPSS software (IBM Corp, 2013 Released).

## Results

### Patient Characteristics

Patient characteristics are presented in Table 2. The LA diameter was significantly larger in the persAF than in the SR group (*p* = 0.016). We included 13 recordings from the 7 patients, with 30–75 s duration (38.34 ± 15.7 s). CV was higher on the endocardial than on the epicardial surface (66 ± 8 vs. 58 ± 6 cm/s, *p* < 0.05).

**TABLE 2:** Patient characteristics.

Patient number	Gender	BMI	Age (years)	Operations	LA-d (mm)	EF (%)	Rhythm
1	Male	23.5	70–75	AVR	45	35	persAF
2	Female	24	70–75	MVR	100	50	persAF
3	Male	30	65–70	CABG	69	60	persAF
4	Male	26.5	75–80	AzVR	72	30	persAF
5	Male	30	70–75	CABG	37	60	SR
6	Male	18.7	70–75	CABG	28	60	SR
7	Male	24.2	60–65	AVR	40	30	SR

*AVR, aortic valve replacement; MVR, mitral valve replacement; CABG, coronary artery bypass graft; LA-d, left atrial diameter in mm; EF, left ventricular ejection fraction; persAF, longstanding persistent AF; SR, sinus rhythm.*

### Basic AF Characteristics in Human Mapping Files and Simulations

**FIGURE 5:**
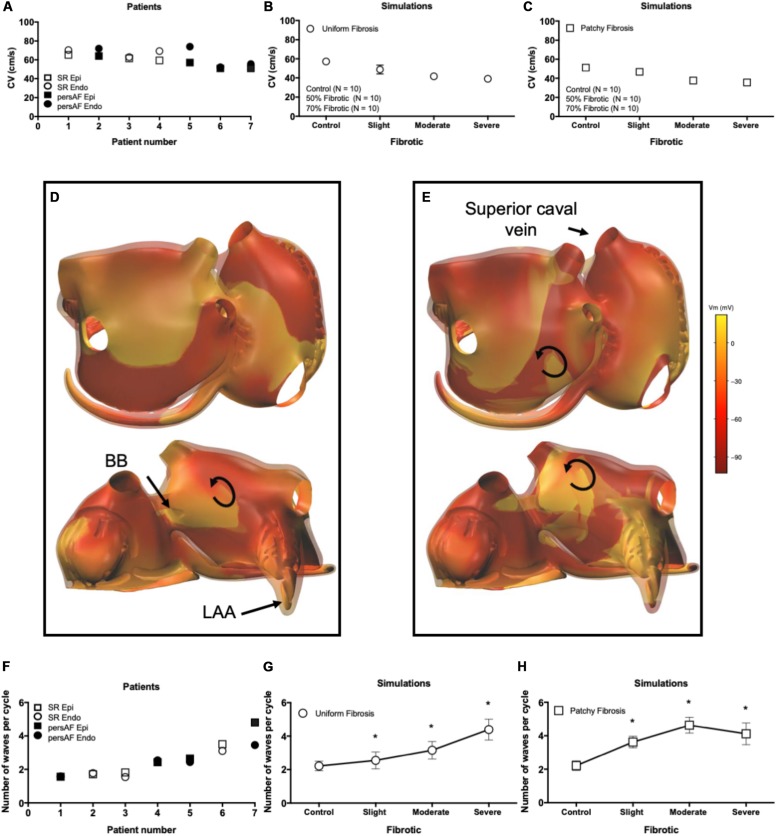
Electrophysiological parameters during AF. **(A)** Conduction velocity (CV) in patients. **(B)** CV in simulations with endomysial (uniform) fibrosis. **(C)** CV in simulations with patchy fibrosis. **(D)** Snapshot of activation in a control simulation. Brighter colors indicate higher transmembrane potentials. The epicardial layer is shown in a semi-transparent manner so that EED can be appreciated. **(E)** Snapshot of a severe fibrotic simulation with patchy fibrosis distribution. **(F)** Number of endocardial and epicardial waves per cycle in patient recordings. **(G)** Number of epicardial waves per cycle in simulations with uniform fibrosis. **(H)** Number of epicardial waves per cycle in simulations with patchy fibrosis. ^∗^indicates significant difference from the control group (*p* < 0.05).

The average AFCL was 140.3 ± 6.1 ms in SR patients and 194.3 ± 3.7 ms in persAF patients. In the simulations, average AFCL ranged from 143.4 ± 2 to 148.7 ± 1.4 ms in control and severe fibrotic tissue, respectively. Endocardial and epicardial CVs were between 50.9 and 74.1 cm/s in human recordings (64 ± 4 for SR patients and 67 ± 23 cm/s for persAF patients), compared to 57.3 ± 3.5, 48.7 ± 4.7, 41.6 ± 1.7, and 39.1 ± 3.4 cm/s in control, slight, moderate, and severe fibrotic simulations with uniform fibrosis pattern (Figures 5A,B). In patchy fibrosis simulations, CVs were 51.2 ± 3.4, 46.7 ± 4.7, 37.6 ± 1.6, and 35.6 ± 1.8 cm/s in control, slight, moderate, and severe fibrotic tissue (Figure 5C).

### AF Conduction Pattern Complexity

Atrial fibrillation conduction pattern complexity was assessed in terms of the number of fibrillation waves in both human recordings and simulations. In Figures 5D,E, examples of fibrillation patterns in control and moderate fibrotic simulations are depicted. Fibrosis increased conduction pattern complexity in the RA, LA anterior wall, and pulmonary vein area.

Quantitative results derived from 7 patients and 70 simulations are shown in Figures 5F–H. In the graphs, patients were sorted and numbered based on increasing complexity. The number of waves in the endocardium and epicardium in patients was similar. The average number of waves per cycle was 2.46 ± 1.11 in SR patients and 3.07 ± 0.91 in persAF patients (Figure 5F). In simulations with uniform fibrosis, the average number of waves per cycle in the epicardium increased significantly from 2.2 ± 0.3 to 2.5 ± 0.5, 3.1 ± 0.5, and 4.4 ± 0.6 in control, slight, moderate and severe fibrotic simulations, respectively. In patchy fibrosis simulations, the average number of waves increased significantly from 2.2 ± 0.2 in control to 3.6 ± 0.3 and 4.7 ± 0.5 in slight and moderate fibrotic simulations, and decreased to 4.1 ± 0.6 in severe fibrotic simulations. In slightly and moderately fibrotic simulations, the average number of waves was higher for patchy fibrosis than for uniform fibrosis (Figures 5G,H).

### EED in Human Mapping Recordings and Simulations

**FIGURE 6:**
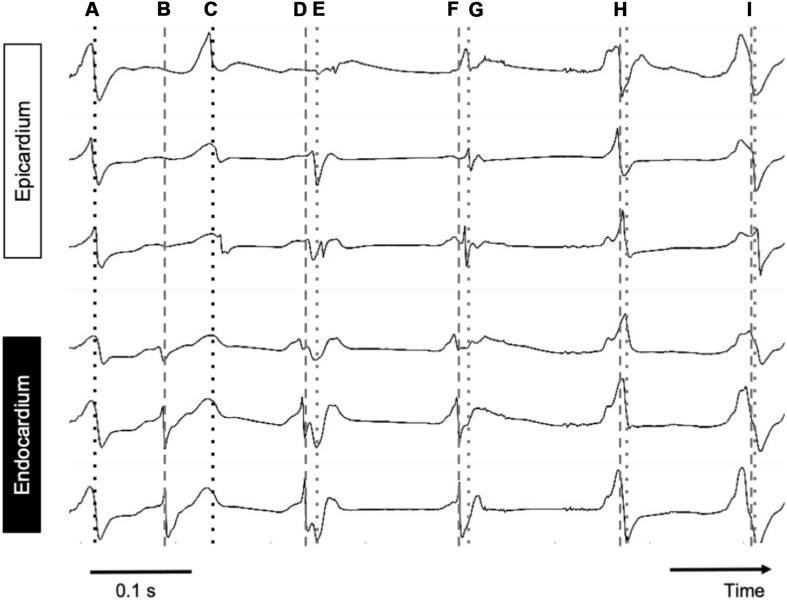
Simultaneous endo–epicardial mapping in a patient’s RA during AF. Dotted line, epicardial activation; dashed line, endocardial activation. Depicted are six simultaneous electrograms, three on each exactly opposing side of the atrial wall. At points A, H, and I, almost synchronous activity was seen with slightly earlier activation at the endocardium, while at B and C, endo- and epicardial activation were out of phase.

Pairs of measured endocardial and epicardial electrograms are illustrated in Figure 6. At the beginning of the recording, endocardial and epicardial deflections occurred simultaneously (“A”) while 80 ms later only the endocardial recordings showed steep deflections. It is likely that at time point “A” the deflections in the endocardial signals reflected far-field potentials from the epicardial layer. From time point “C” onward the deflections occurred nearly simultaneously again in both layers.

**FIGURE 7:**
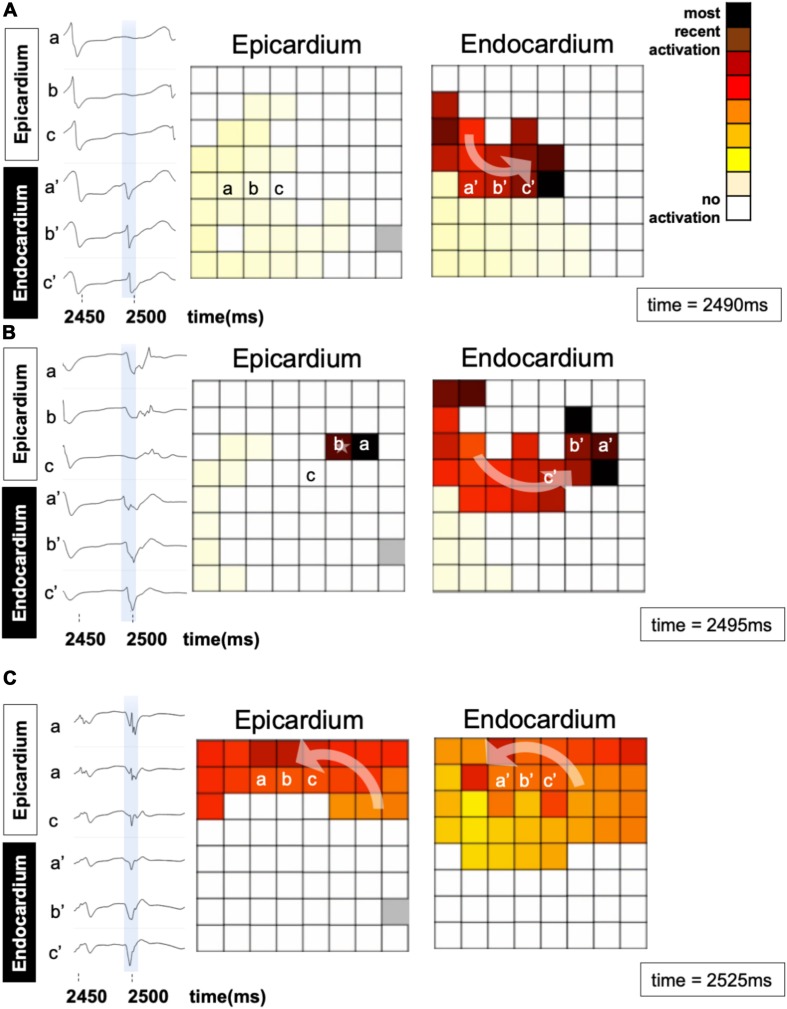
An example of a breakthrough occurring in the epicardial layer in a patient. **(A)** A wave enters the endocardial layer (red) (a′, b′, and c′). The epicardium is not activated (a, b, c). The left panels show the local electrograms. The right panels show simultaneous endo–epicardial activation maps at three different time instants. **(B)** Epicardial breakthrough (b) resulting from propagation from the endocardium. **(C)** The breakthrough in the epicardial layer spreads further in synchrony with the endocardium.

An epicardial BT recorded in a patient is depicted in Figure 7. A fibrillation wave (red) entered the endocardial layer (a′, b′, c′ in panel A) while the epicardial layer was not activated (a, b, c). Five milliseconds later, an epicardial BT appeared (panel B). During the next 30 ms the wave spread simultaneously on both sides (panel C).

**FIGURE 8:**
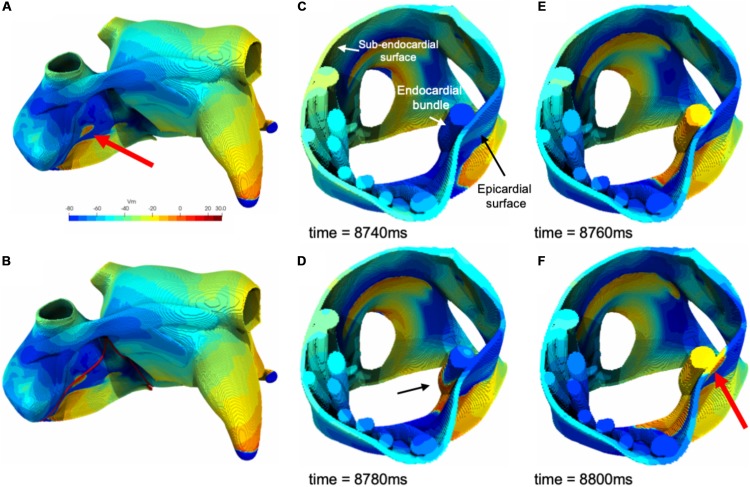
**(A)** An example of a simulated epicardial breakthrough indicated by a red arrow. **(B)** Red lines indicate clipping planes. **(C)** Clipped right atrium at the breakthrough location. **(D)** A fibrillation wave propagating through an endocardial bundle (black arrow). **(E)** The fibrillation wave propagated transmurally from the endocardial bundle to the epicardial surface. **(F)** Appearance of the epicardial breakthrough (red arrow).

In Figure 8 an example of epicardial BT in the model is illustrated. As illustrated in this figure a fibrillation wave entered and propagated in an endocardial bundle while the opposing epicardial layer was quiescent (Figures 8C,D). After 20 ms the wave propagated transmurally and appeared as an epicardial BT (Figures 8E,F).

**FIGURE 9:**
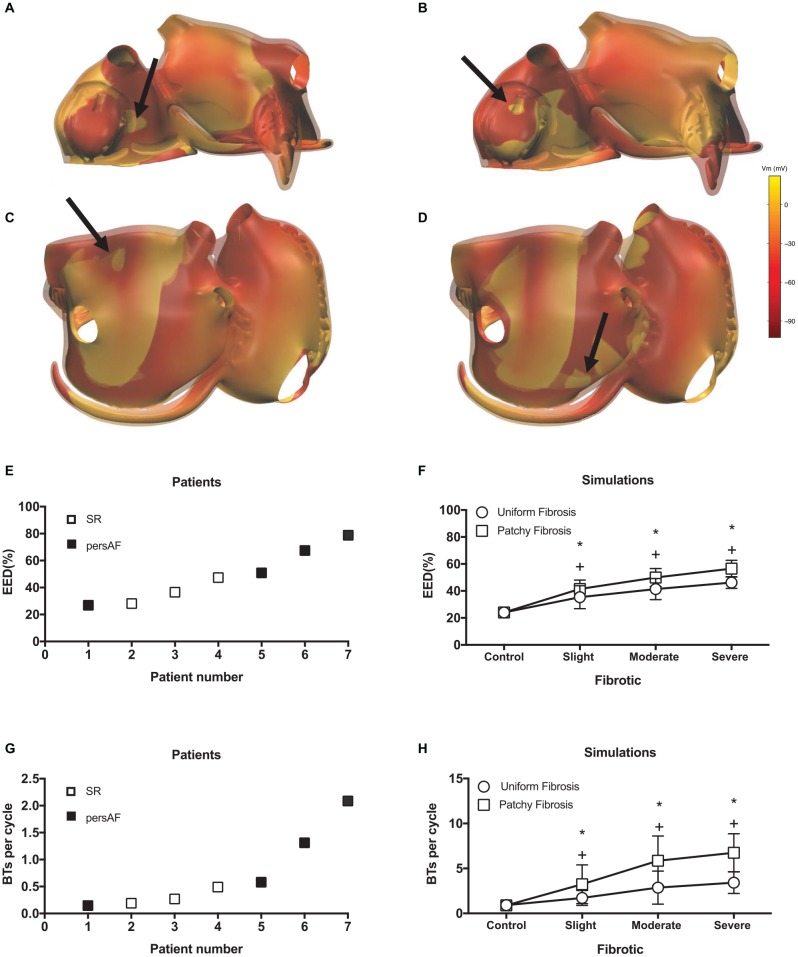
Examples of simulated BTs. Snap shots of BTs in **(A)** a control simulation, **(B)** a moderate fibrotic simulation with patchy fibrosis pattern, **(C)** a severe fibrotic simulation with uniform fibrosis, and **(D)** a severe fibrotic simulation with patchy fibrosis. The colorbar indicates the transmembrane voltage. **(E)** EED in patient recordings. **(F)** EED in simulations. *indicates significant difference from control in patchy fibrosis (*p* < 0.05) and ^+^ indicates significant difference from control in uniform fibrosis (*p* < 0.05). Number of BTs per cycle in panel **(G)** in patient recordings and **(H)** simulations.

Additional examples of BTs in simulations are shown in Figure 9. While most BTs occurred in areas overlying endocardial bundles (Figures 9A,B), some were seen in areas without underlying bundles (Figures 9C,D).

Both in the patient recordings and in the simulations, the degree of EED ranged between 20 and 80%. In the patients, there was a trend toward more EED in the persAF group (Figure 9E). In the simulations, fibrosis increased the degree of EED (Figure 9F) significantly from 24.1 ± 3.4 to 35.4 ± 8.5, 41.4 ± 7.8, and 46.2 ± 4.3% in control, slight, moderate, and severe uniform fibrotic models. In patchy fibrosis simulations, EED increased significantly from 24.1 ± 3.4 to 41.5 ± 6.6, 49.9 ± 6.7, and 56.58 ± 6.2%, for control, slightly, moderately, and severely fibrotic models.

The number of BTs per cycle increased significantly from 0.92 ± 0.55 in control to 3.42 ± 1.2 in severe uniform and 6.74 ± 2.11 in severe patchy fibrotic models (Figure 9H). The numbers in the simulations were higher than in patients (Figure 9G), because they were counted over the entire atrial surface in simulations and within the recording area in patients.

**FIGURE 10:**
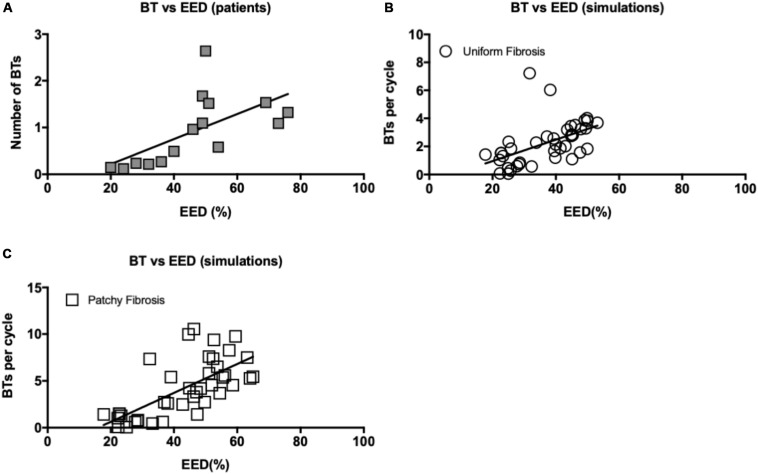
**(A)** Correlation between EED and BT incidence in patient recordings. Pearson’s correlation *r* = 0.61 for both endo- and epicardial BT, *p* < 0.05. **(B)** Correlation between EED and breakthrough incidence in uniform fibrosis (*r* = 0.51, *p* < 0.05) and **(C)** patchy fibrosis simulations (*r* = 0.69, *p* < 0.05).

Both in patient recordings and in simulations, there was a positive correlation between the average number of BTs per cycle and the degree of EED (Pearson’s correlation *r* = 0.61 and *p* < 0.05, Figure 10A for the patient recordings, *r* = 0.51, *p* < 0.05, Figure 10B for the uniform fibrosis simulations, and *r* = 0.69, *p* < 0.05, for the patchy fibrosis simulations, Figure 10C).

## Discussion

We developed a realistic atrial model that displays EED and transmural conduction, closely resembling the human 3D substrate for AF. This study shows that atrial fibrosis in the epicardial layer can increase EED of electrical activity and incidence of BTs to a comparable degree as in patients. In simulations as well as in patient recordings, BTs contributed significantly to AF complexity. Importantly, recent high-density mapping studies in human AF have demonstrated that around 30% of all fibrillation waves propagate from the epicardial layer to the endocardial bundle network or vice versa (de Groot et al., 2010, 2016). Our study shows that different patterns of fibrosis, patchy, and uniform can produce different degrees of AF complexity, in terms of number of waves, BTs, and EED.

### Consequences of Our Study

Two of the most enigmatic observations in human AF are the increased EED, which develops in the thin atrial wall, and the occurrence of BTs contributing to AF stability (de Groot et al., 2010, 2016; Eckstein et al., 2011; Verheule et al., 2014). EED of electrical activity has been described by Schuessler et al. (1993) in isolated canine RA using simultaneous endo–epicardial mapping. More recently, optical mapping in isolated RA walls from explanted human hearts showed that intramural micro-reentry circuits may drive AF (Hansen et al., 2015). A sheep model showed clear evidence of endo–epicardially dissociated activity and BT waves in the LA (Gutbrod et al., 2015). de Groot et al. (2016) confirmed this with contact endo–epicardial mapping in human RA during cardiac surgery, with 65% of BTs explained by activation on the opposing layer. This agrees with our findings in human RA where a higher degree of EED correlated with a larger number of BTs resulting in a 3D conduction pattern that enhances the overall complexity of AF (Verheule et al., 2014).

Nevertheless, the precise relation of EED occurrence and increased BT incidence as a pathophysiological mechanism is not well understood. A previous modeling study has shown that disruption of electrical coupling between the endocardial and epicardial layers increased EED and BT rate (Gharaviri et al., 2017). This would be equivalent to extensive mid-wall fibrosis, but there is little evidence for such a phenomenon in animal models or AF patients. In a recent study, Verheule et al. (2013) compared histological changes after 6 months of AF with 2 weeks of AF in a goat model of persAF. Notably, next to uniform myocyte hypertrophy, prolonged AF led to significant endomysial fibrosis (also called interstitial or “reactive” fibrosis) concentrated in the outer layer of the atrial wall as opposed to endocardial trabeculae. These changes were associated with impaired transverse propagation within the epicardial layer and contributed to complex fibrillation patterns and AF stability. In our highly detailed computer model, we simulated two patterns of fibrosis, uniform and patchy, in the atrial wall. We found that, indeed, increased fibrosis led to enhanced EED and BT. There was a trend toward more complex patterns in patchy fibrosis. Although both fibrosis patterns were artificial, our overall findings correlated well with the results derived from endo–epicardial mapping in human RA.

### Comparison to Other Computer Models

Numerous modeling studies have been performed to understand the mechanisms underlying AF. Models developed for this purpose can be divided into (1) surface models, which treat the atria as a two-dimensional sheet folded into the shape of the atria (Haissaguerre et al., 2007; Uldry et al., 2012) and (2) volumetric models that incorporate regional variation in wall thickness and fiber orientation (Vigmond et al., 2001; Gong et al., 2007; Aslanidi et al., 2011; Colman et al., 2013; Krueger et al., 2013). A few studies have investigated the effect of atrial anatomy, wall thickness, and endocardial bundles (modeled as higher local wall thickness) on fibrillation patterns and AF maintenance (Aslanidi et al., 2011; Krueger et al., 2013; Zhao et al., 2017). Recently, Labarthe et al. (2014) investigated the effect of transmural heterogeneities in the atria using a bilayer model. However, none of these models showed transmural conduction of fibrillation waves and BTs. The novelty of our model is that, due to the presence of a trabecular network, realistic wall thickness, layered fiber bundles, and a sufficiently high spatial resolution (200 μm), it allows us to investigate these phenomena, and to assess the effect of structural remodeling in this context.

The fiber orientations integrated in our model were based on histological studies (Ho et al., 2002; Ho and Sanchez-Quintana, 2009; Pashakhanloo et al., 2016). Importantly, based on sub-millimeter resolution diffusion tensor MRI, Pashakhanloo et al. (2016) reported that the main features of fiber orientations were well preserved among subjects. The preferential fiber directions described in their study, such as LA posterior and anterior wall, RA free wall, LAA, and circumferential fiber orientations close to PVs and orifices are well reflected in our 3D model.

The properties for the material present in the atria were set to produce an approximately normal P wave in case of SR. As previously discussed by Potse (2019), the surface-to-volume ratio used in our simulation was larger than measured values because otherwise a realistic CV cannot be achieved. This is a well-known problem. In previous studies we often used a value of 1000 cm^−1^. In this case we used 800 cm^−1^ to account for the fact that the MRI subject was a large adult man with a proportionally large heart (Potse et al., 2016).

In this study, we simulated the effect of two different patterns of fibrosis, patchy and uniform, on EED and transmural conductions. To generate patchy fibrosis patterns, we generated a spatially correlated, anatomy-tailored random field. A similar procedure for generating spatially correlated fibrosis was also proposed by Clayton (2018), but on a 2D tissue. In this work, we also account for the topology of the atria, and fibrosis is generated in such a way that geometrically close but anatomically distinct regions will not be correlated as they would just by considering the Euclidean distance.

The role of fibrosis in AF initiation and maintenance has also been investigated with computational studies (McDowell et al., 2015; Zhao et al., 2015). McDowell et al. (2015) reported that AF was only induced and sustained in atrial models with higher degrees of fibrosis. It has also been shown that the spatial distribution of fibrosis modulates AF dynamics (Tanaka et al., 2007; McDowell et al., 2013, 2015), suggesting that the unique distribution of atrial fibrosis in each patient may govern the location of AF rotors (McDowell et al., 2015). Roy et al. (2018) illustrated that atrial wall thickness, as well as fibrosis patterns, can affect the location of AF rotors in atria. In a study by Zahid et al. (2016) fibrosis distributions through atrial walls were measured using an adaptive histogram thresholding algorithm in LGE-MR images. It was shown in this study that reentrant drivers perpetuating AF persist in regions with a high degree of intermingling between fibrotic and non-fibrotic tissue. Zhao et al. (2017) assessed spatial patterns of fibrosis and myofiber architecture in an *ex vivo* pair of human atria, in high resolution, using contrast-enhanced MRI (CE-MRI) and showed that AF reentrant drivers were distinguishable by fingerprints of specific intermediate wall thickness and fibrosis combined with twisted myofiber orientation. Our study focuses on the occurrence of 3D conduction and BT, which likely also have a strong role in AF perpetuation. Here, we demonstrate that epicardial fibrosis is sufficient to provoke EED and BT and that EED is highly correlated to the BT rate supporting the view that EED is an important driving force for BTs to occur.

### Limitations

Our clinical mapping data were restricted to the right atrial free wall, largely for safety reasons. However, in a goat model, we did not observe qualitative differences in EED of electrical activity between right and left atria (Eckstein et al., 2011).

The clinical study was performed in a small number of patients. This is mainly due to the complex and difficult procedure of simultaneous endo–epicardial mapping during surgery. These data were used to demonstrate that the modeling results are quantitatively comparable to the situation in patients.

The number of BTs per cycle observed in the model was larger than in patients, but not as large as could be expected based on the observed area (180 cm^2^ in the model, and only 1 cm^2^ in patients). This is partially explained by the fact that the clinical observations were limited to a trabeculated area, where BT incidence would be expected to be larger. In contrast, in the modeling study, BTs were identified and quantified in the entire atrial wall.

In this study, fibrosis patterns were based on a mathematical algorithm and not on clinical imaging data. It could be of interest to repeat these simulations with patient-specific fibrosis patterns, enhanced with an algorithmically determined fine-grain pattern that would reflect the results of histological studies.

The electrophysiological manifestation of fibrosis is multifaceted including uncoupling of cells, alterations of ionic channels, and changes in myocyte-(myo)fibroblast coupling (Roney et al., 2016). However, in this study we only included the reduction in cell–cell coupling as representation of fibrosis. We modeled fibrosis by setting the conductivity to zero exclusively in the cross-fiber direction in a variety of percentages of cells reflecting the notion of transverse but not longitudinal conduction delays in aged and more fibrotic atrial muscle (Spach and Dolber, 1986). An alternative would be to assign “fibrosis” and “fibroblast” properties to specific model elements. This would allow to match the fibrosis content with clinical data (Roney et al., 2016; Zahid et al., 2016), which is not possible with our method. However, this would also result in an unrealistically coarse grain of fibrosis, as this is determined by the size of the model elements, i.e., 200 μm.

To avoid confounding factors, we deliberately did not implement heterogeneity in ionic parameters in our model.

In this study we only used a single atrial geometry. However, inter-subject variability in atrial geometries and fibrosis distribution play an important role in AF perpetuation.

Directional differences in CV in the simulated AF episodes were not quantified. Therefore, conduction vectors with low CV (conduction block) were not excluded for CV calculations. Hence, calculated CV in simulated AF episodes were lower than the measured CV in clinical recordings. In addition, for the simulated AF only epicardial CV was determined, while in clinical recordings both endocardial and epicardial CV were calculated.

Finally, we did not consider ectopic focal discharges as drivers for AF. However, in a previous modeling study we have shown that the relation between EED and BT rate does not critically depend on whether AF is driven by reentry or by ectopic discharges (Gharaviri et al., 2017).

## Conclusion

We developed the first computer model for AF that includes an epicardial layer and endocardial bundle network with realistic assumptions on fiber directions and can simulate three-dimensional propagation of fibrillation waves similar to patterns observed in clinical mapping studies. Simultaneous high-density endo–epicardial mapping in patients confirmed the validity of the main quantitative characteristics of AF and EED in this model. Modeling results showed that isolated epicardial fibrosis can explain increased EED of electrical activity and BT incidence in a complex substrate for AF.

Our findings may have major clinical implications for the interpretation of mapping results to identify targets for AF ablation. Our study demonstrates that conduction patterns recorded on the epicardial or endocardial surface of the atrium do not necessarily reflect the overall conduction pattern in the atrial wall.

## Data Availability Statement

The datasets generated for this study are available on request to the corresponding author.

## Ethics Statement

The studies involving human participants were reviewed and approved by fhml-rec@maastrichtuniversity.nl. The patients/participants provided their written informed consent to participate in this study.

## Author Contributions

US, SV, and MP designed the study. AG and MP carried out the simulations. MP and SP developed and validated the computer model. MP and SP provided illustrations. AG collected and analyzed simulation data. EB and JM collected clinical data. EB analyzed clinical data. SZ provided software for analyzing clinical data. AG wrote the manuscript. US, SV, MP, AA, and RK provided scientific inputs and Interpreted the results. All authors contributed in revising the work, approved the final version to be published, and agreed to be accountable for all aspects of the work.

## Conflict of Interest

US is co-founder and shareholder of YourRhythmics BV, a spin-off company of the University Maastricht. He holds intellectual property with Roche and YourRhythmics BV. He received consultancy fees or honoraria from Johnson & Johnson, Roche Diagnostics (Switzerland), and Bayer Healthcare (Germany). AA is a consultant to Boston Scientific, Backbeat, Biosense Webster, Cairdac, Corvia, Microport CRM, Philips, and Radcliffe Publisher. He received speaker fee from Boston Scientific, Medtronic, and Microport. He participates in clinical trials sponsored by Boston Scientific, Medtronic, and Philips. He has intellectual properties with Boston Scientific, Biosense Webster, and Microport CRM. The remaining authors declare that the research was conducted in the absence of any commercial or financial relationships that could be construed as a potential conflict of interest.
