# Supplementary material for: Epicardial Fibrosis Explains Increased Endo–Epicardial Dissociation and Epicardial Breakthroughs in Human Atrial Fibrillation
Source: Front Physiol. 2020 Feb 21;11:68. doi: 10.3389/fphys.2020.00068 (PMC7047215; doi:10.3389/fphys.2020.00068)
Supplement: Supplementary file 1 [file Data_Sheet_1.pdf]

Supplemental Material for Epicardial Fibrosis Explains Increased Endo-Epicardial Dissociation and Epicardial Breakthroughs in Human Atrial Fibrillation

Ali Gharaviri, PhD; Elham Bidar, MD; Mark Potse, PhD; Stef Zeemering, PhD; Sander Verheule, PhD; Simone Pezzuto, PhD; Rolf Krause, PhD; Jos G. Maessen, MD, PhD; Angelo Auricchio, MD, PhD; and Ulrich Schotten, MD, PhD

S1 Diffuse and patchy fibrosis model

Fibrotic tissue was simulated by randomly assigning the special type "fibrotic" to a given fraction f of model elements. In the diffuse fibrosis model, we simply took a random combination of $\lfloor N\, f \rfloor$ myocardial voxels, where N is the total number of myocardial voxels. We simulated 3 diffuse fibrosis models, with 50%, 70%, and 80% fraction of fibrosis.

Patchy fibrosis models were based on a heterogeneous distribution of the probability of a voxel being selected as "fibrotic". The background probability distribution was a multiscale, spatially-correlated random field (see Figure S1, panel E and F). This random field was obtained with the following steps:

1. Two random Gaussian fields, $g_1(x)$and $g_2(x)$, with squared-exponential, spatial covariance of the form

$$k(x,y) = \exp\left(-\frac{d(x,y)^2}{2\rho^2}\right), \quad x,y \in \Omega, \tag{1}$$

   of characteristic length ρ respectively equal to 2 cm and 4mm were generated. The fields $g_1(x)$and $g_2(x)$ account respectively for coarse-scale and fine-scale spatial correlations. The function $d(x,y)$ was the geodetic distance between the points x and y, computed numerically with the Eikonal equation.

2. The random fields g_1 and g_2 were averaged into a single field g:

$$g(x) = \frac{g_1(x) + g_2(x)}{2}.$$

3. The resulting random field was rescaled as follows:

$$h(x,t) = \frac{1}{2}\left(1 + \tanh\left(\frac{g(x) - t}{\varepsilon}\right)\right),$$

   with $\varepsilon = 0.2$ and $t \in= [0,1]$.

4. For each voxel of the computational grid, voxel x was fibrotic if $h(x,t) \geq H$, $H \sim \mathcal{U}([0,1])$, with t such that the total number of fibrotic voxels was, on average, equal to the given fraction f:

$$\int_{\Omega} h(x,t) = f|\Omega|.$$

The coarse-scale random field $g_1(x)$ was obtained from the truncated Karhuhen -Loève (KL) expansion of the covariance function. The method is outlined by Pezzuto et al. (2018) Briefly, a low-rank approximation of the Hilbert-Schmidt operator

$$\mathcal{K}[\Phi](y) = \int_{\Omega} k(x,y)\Phi(\mathrm{x})d_x,$$

associated to the covariance function (1), was considered. The distance $d(x,y)$ inside the kernel was evaluated with the eikonal equation. Since the correlation function decays slowly, because the correlation length is large, only a few terms in the KL expansion were needed.

The fine-scale random field $g_2(x)$ was sampled through the iterative solution of a stochastic partial differential equation (SPDE) of the following form (Lindgren et al., 2011):

$$(\theta^2 - \Delta)^{\alpha/2} u(x) = \mathcal{W}(x), \; x \in \Omega \qquad (2)$$

with $\alpha = v + d/2$, $v > 0, \theta > 0, \Delta$ the Laplace operator, and $\mathcal{W}$ Gaussian white noise with unit variance. In the model, $d = 3$ (space dimension), and $\kappa^2 = 4v/\rho^2$. With these assumptions, when $v \to \infty$, the solution $u(x)$approximates a random Gaussian field with square-exponential Gaussian correlation with characteristic length ρ. The parameter v was set equal to $\frac{4m-3}{2}, m = 1,2, \dots$, so that the pseudo-differential operator $(\theta^2 - \Delta)^{\alpha/2}$ was the product of m elliptic operators of the form $(\theta^2 - \Delta)$. The problem (2) was eventually solved iteratively as follows:

$$u_0 = \mathcal{W}, \qquad (\theta^2 - \Delta)u_{m+1} = u_m, \qquad m = 1,2, \dots \qquad (2)$$

An example of diffuse and patchy fibrosis is shown in Figure S1.

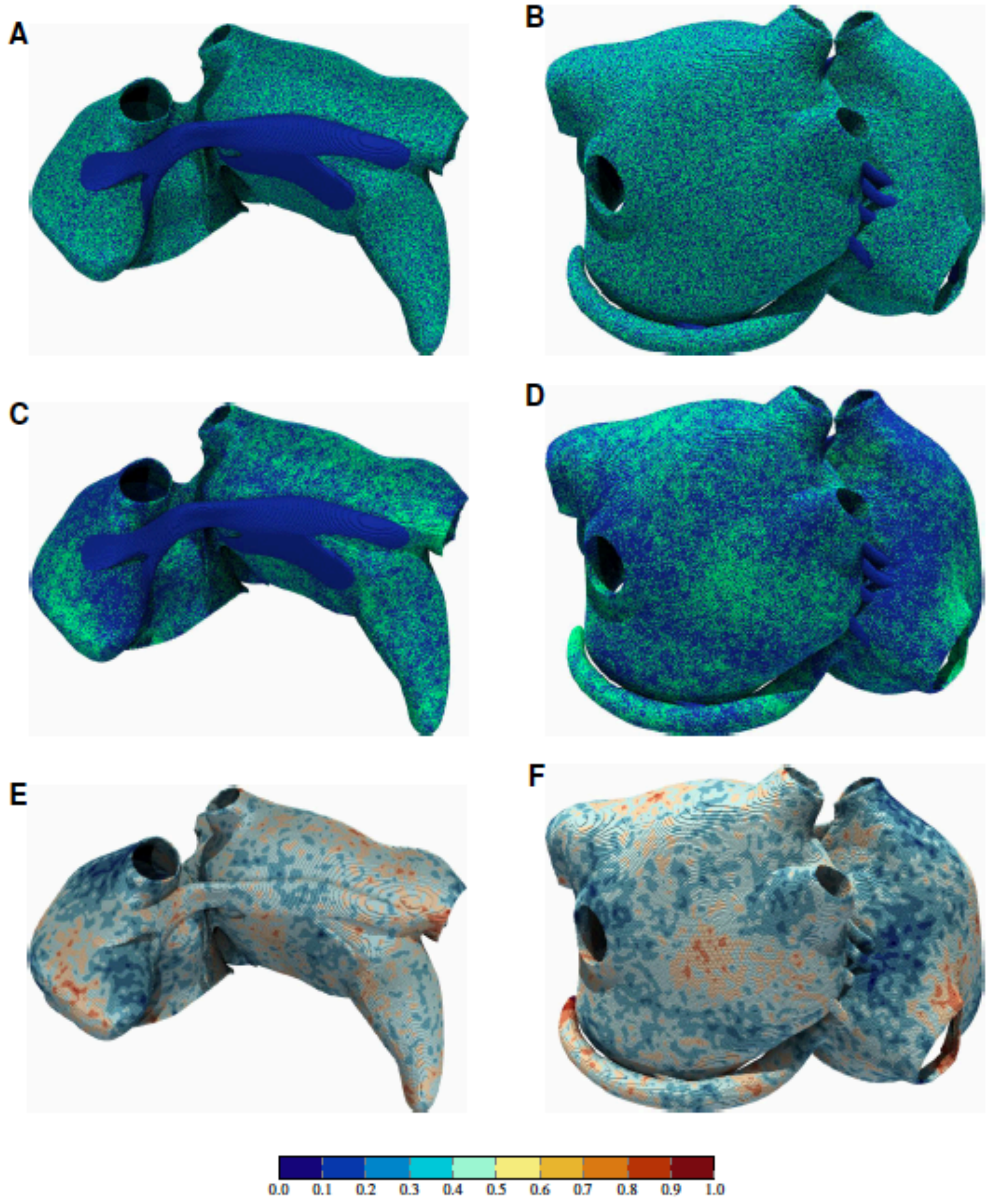

Figure S1: Posterior and anterior view of the atria with diffuse and patchy fibrosis. A) Diffuse fibrosis model, 50% fibrotic (anterior view). B) Diffuse fibrosis model, 50% fibrotic (posterior view). C) Patchy fibrosis model, 50% fibrotic (anterior view). D) Patchy fibrosis model, 50% fibrotic (posterior view). E) Background random field for patchy fibrosis (anterior view). F) Background random field for patchy fibrosis (posterior view). Brighter voxels are fibrotic in the model.

A

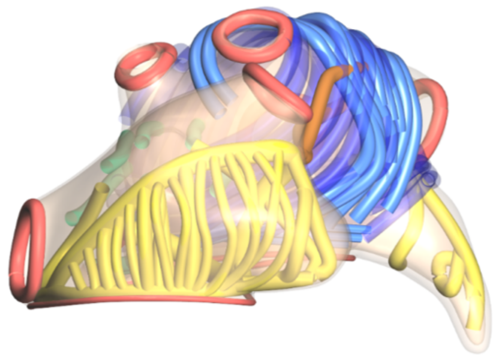

B

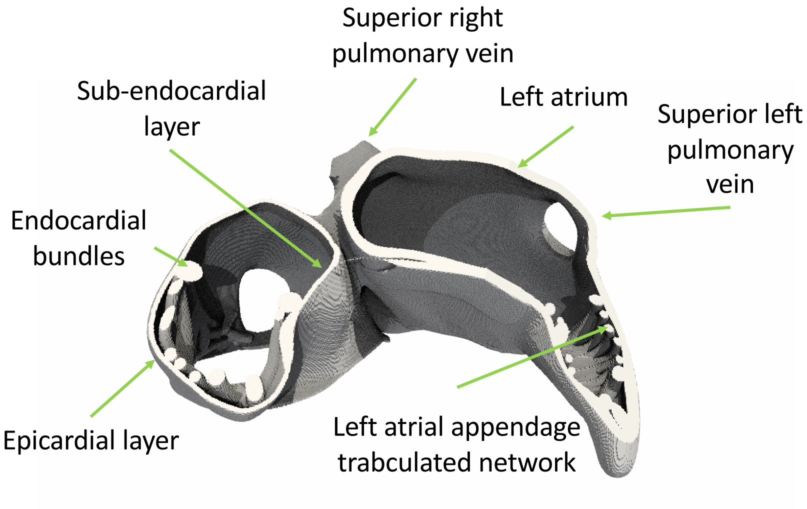

Figure S2: A) Geometrical objects created manually. Endocardial bundles are in yellow. Splines used for fiber orientation generations are in Blue and dark blue. B) Cross sectional view of the atrial geometry.

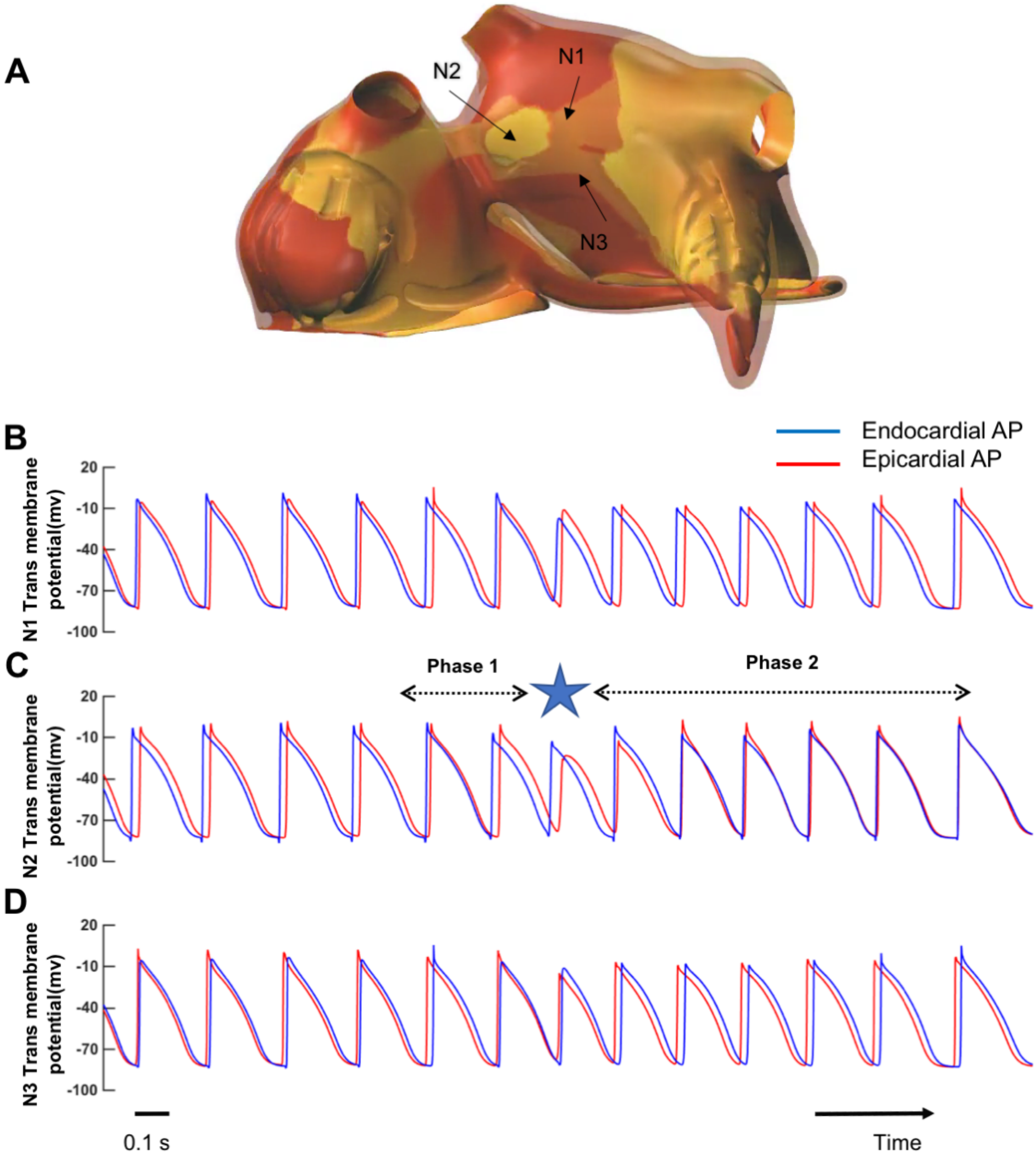

Figure S3: Simultaneous endocardial and epicardial simulated action potentials during a breakthrough. A) An example of a breakthrough and the locations of the simultaneous endo-epi APs. B) Simultaneous endo-epi AP at a neighbouring node close to the position of the breakthrough (node N1). C) Simultaneous endo-epicardial APs at the centre of a breakthrough (N2). The star indicates the moment of epicardial breakthrough (node N1). Phase 1 indicates time period in which EED started to increase, leading to a breakthrough. Phase 2 illustrates the disappearance of EED due to the occurrence of the breakthrough. D) Simultaneous endo-epi AP at a node further from the BT position (node N3).

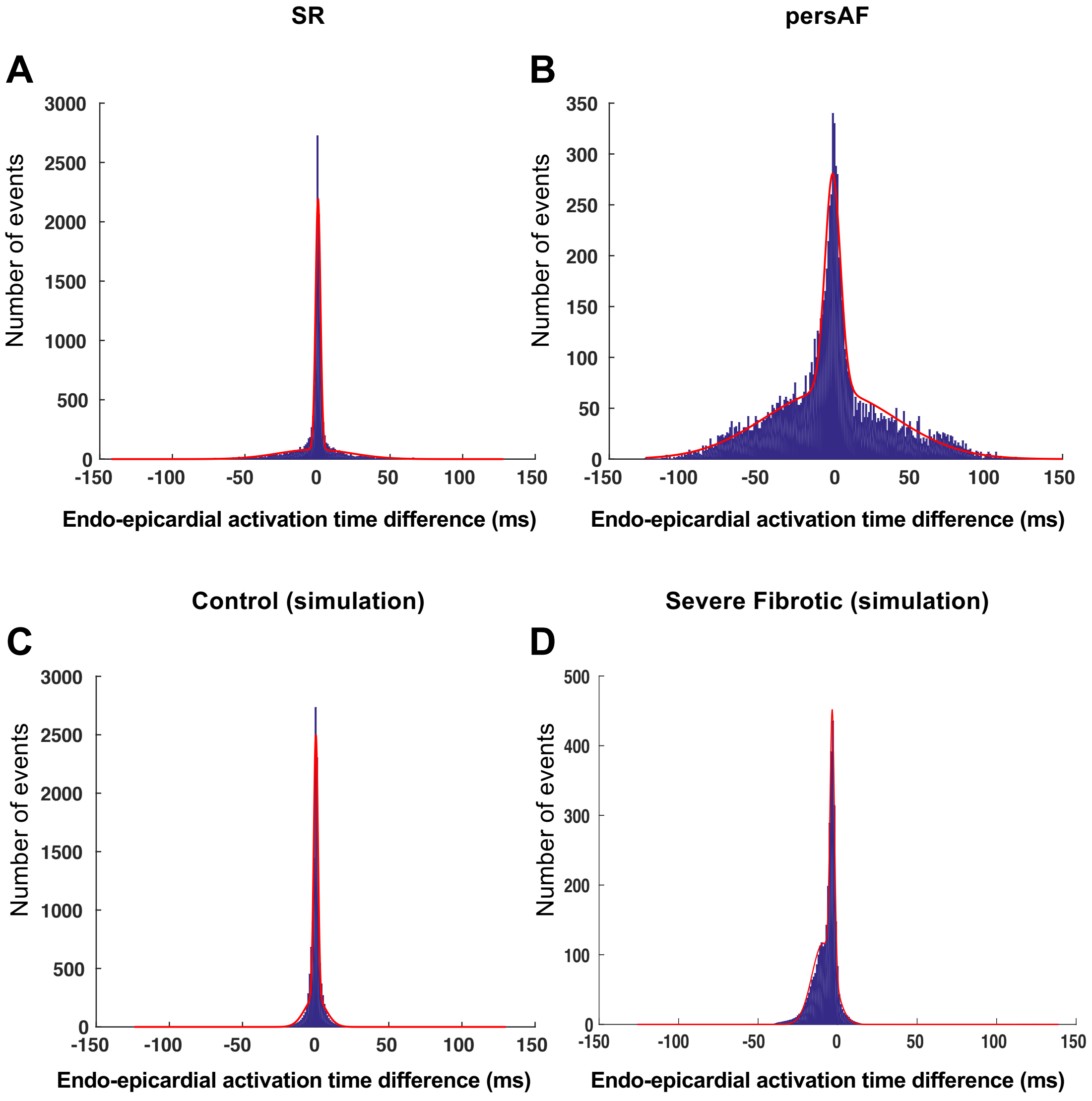

Figure S4: Activation time differences between the endo- and epicardial layers in patients (upper panels) and simulations (lower panels). A) Histogram of endo-epicardial activation time differences in A) an SR patient, B) a persAF patient, C) a control simulation and D) a simulation of severe fibrosis. X-axes denotes the difference in activation time (ms) and y-axes denotes the number of events.

Reference:

Lindgren, F., Rue, H., and Lindström, J. (2011). An explicit link between Gaussian fields and Gaussian Markov random fields: the stochastic partial differential equation approach. *Journal of the Royal Statistical Society: Series B (Statistical Methodology)* 73(4), 423-498. doi: 10.1111/j.1467-9868.2011.00777.x.

Pezzuto, S., Gharaviri, A., Schotten, U., Potse, M., Conte, G., Caputo, M.L., et al. (2018). Beat-to-beat P-wave morphological variability in patients with paroxysmal atrial fibrillation: an in silico study. *Europace* 20(suppl_3), iii26-iii35. doi: 10.1093/europace/euy227.
